# Bardoxolone Methyl Ameliorates Myocardial Ischemia/Reperfusion Injury by Activating the Nrf2/HO-1 Signaling Pathway

**DOI:** 10.1155/2023/5693732

**Published:** 2023-02-22

**Authors:** Anwu Huang, Zhaolin Wang, Hua Tang, Zhuyin Jia, Xiaojun Ji, Xuehua Yang, Wenbing Jiang

**Affiliations:** ^1^Department of Cardiology, Wenzhou Central Hospital, The Second Affiliated Hospital of Shanghai University, Wenzhou, Zhejiang, China; ^2^Institute of Translational Medicine, Shanghai University, Shanghai, China; ^3^Department of Cardiology, Shanghai Zhongye Hospital, Shanghai, China

## Abstract

**Background:**

Myocardial ischemia/reperfusion (I/R) injury is a severe heart problem resulting from restoring coronary blood flow to the myocardium after ischemia. This study is aimed at ascertaining the therapeutic efficiency and action mechanism of bardoxolone methyl (BARD) in myocardial I/R injury.

**Methods:**

In male rats, myocardial ischemia was performed for 0.5 h, and then, reperfusion lasted for 24 h. BARD was administrated in the treatment group. The animal's cardiac function was measured. Myocardial I/R injury serum markers were detected via ELISA. The 2,3,5-triphenyltetrazolium chloride (TTC) staining was used to estimate the infarction. H&E staining was used to evaluate the cardiomyocyte damage, and Masson trichrome staining was used to observe the proliferation of collagen fiber. The apoptotic level was assessed via the caspase-3 immunochemistry and TUNEL staining. Oxidative stress was measured through malondialdehyde, 8-hydroxy-2′-deoxyguanosine, superoxide dismutase, and inducible nitric oxide synthases. The alteration of the Nrf2/HO-1 pathway was confirmed via western blot, immunochemistry, and PCR analysis.

**Results:**

The protective effect of BARD on myocardial I/R injury was observed. In detail, BARD decreased cardiac injuries, reduced cardiomyocyte apoptosis, and inhibited oxidative stress. For mechanisms, BARD treatment significantly activates the Nrf2/HO-1 pathway.

**Conclusion:**

BARD ameliorates myocardial I/R injury by inhibiting oxidative stress and cardiomyocyte apoptosis via activating the Nrf2/HO-1 pathway.

## 1. Introduction

Acute myocardial infarction (AMI) is the primary reason for death and disability, and its incidence rate is sharply increasing in recent years [1]. It is estimated that over 7 million new cases are diagnosed as AMI each year worldwide, among which 800,000 patients need to be hospitalized [2]. In the past years, there were more than 2.4 million AMI-caused deaths in the USA, and more than 4 million deaths in Europe and South Asia [3]. Timely and effective revascularization using percutaneous coronary intervention and thrombolysis is essential for the treatment of AMI [4]. However, this process can further exacerbate myocardial injury, resulting in reperfusion arrhythmia, myocardial stunning, microcirculation disturbance, and lethal reperfusion injury [5]. Although myocardial I/R injury has been recognized for decades, there is still no effective clinical treatment to prevent myocardial I/R injury [6]. Therefore, new therapies are needed to improve the prognosis of patients with AMI.

Accumulating evidence indicates that oxidative stress is the main mechanism underlying myocardial injury [7]. Excessive reactive oxygen species (ROS) productions disrupt the balance between oxidative and antioxidant systems, eventually leading to cell apoptosis, necrosis, and inflammation [8]. Nrf2, mainly maintained in the cytoplasm, is a key transcription factor that plays an important role in regulating oxidation. Public data evidenced that Nrf2 can be activated by oxidative stress to combat ROS and toxic metabolites by increasing the expression of downstream genes. HO-1, as an antioxidant enzyme activated by Nrf2, can resist oxidative damage, regulate apoptosis, and reduce inflammation [9]. Recently, numerous reports have shown that the Nrf2/HO-1 pathway exerts crucial functions in protecting different organs against injury caused by I/R, including in the lung [10], neuron [11], and kidney [12]. More importantly, the protective role of the Nrf2/HO-1 pathway in myocardial I/R injury has been also uncovered. For example, Fullerol alleviates myocardial I/R injury by reducing inflammation and oxidative stress in cardiomyocytes via activating the Nrf2/HO-1 pathway [13]. Therefore, activating the Nrf2/HO-1 pathway is considered as a therapeutic approach to myocardial I/R injury.

Bardoxolone methyl (BARD) is one of the most important drugs to combat oxidative stress by upregulating Nrf2 activity [14]. In phase 3 clinical trials, BARD was used to treat diabetic kidney disease [15] and Alport's syndrome [16]. These trials demonstrated that BARD is a considerable success in treating kidney injury. However, the therapeutic efficacy of BARD on myocardial I/R injury is still elusive. In our study, the effects and action mechanism of BARD in myocardial I/R injury have been preliminarily explored, indicating that BARD can upregulate the Nrf2/HO-1 pathway to attenuate myocardial I/R injury.

## 2. Material and Methods

### 2.1. Animals

At the Institute of Translational Medicine, Shanghai University, male Sprague Dawley rats (200-250 g) were housed at 22-24°C, with free food and water. Before the experimental procedure, the rats underwent acclimatization for one week. This experiment was approved by the Ethical Committee of Shanghai University.

**Figure 1:**
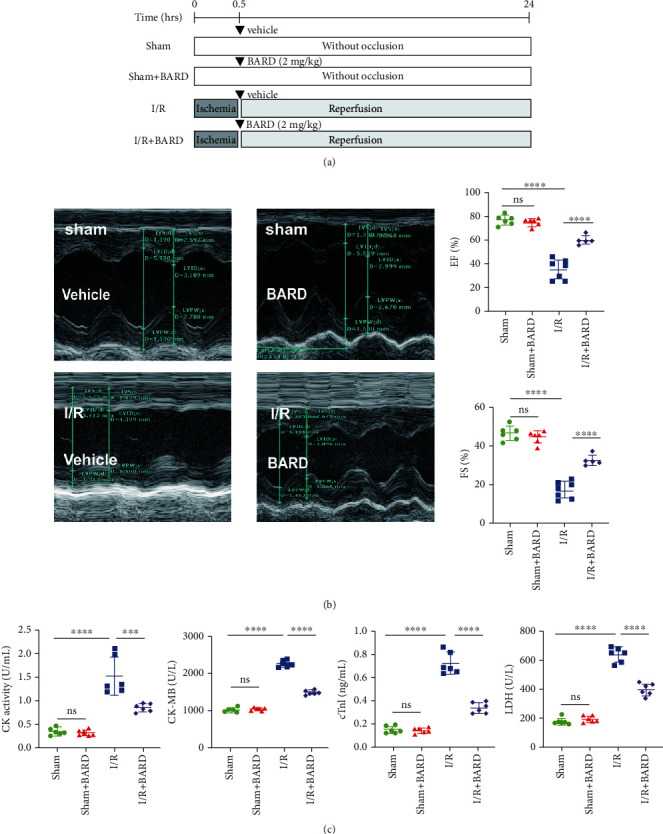
Bardoxolone methyl relieves myocardial I/R-induced cardiac impairment. (a) A scheme showing the timeline of surgical intervention and drug treatment in myocardial I/R rats. (b) Echocardiography was performed to examine the effects of BARD treatments on cardiac functions (*n* = 5 − 7). (c) Myocardial I/R injury serum markers (*n* = 6). ^∗∗∗^*P* < 0.001 and ^∗∗∗∗^*P* < 0.0001. ns: not significant.

Subsequently, rats were assigned into four groups randomly: sham plus vehicle (1% DMSO with PBS), sham plus BARD (2 mg/kg), I/R plus vehicle (1% DMSO with PBS), and I/R plus BARD (2 mg/kg). As previously described, the myocardial I/R injury model was established [17]. In brief, rats were anesthetized and ventilated at a rate of 80 breaths/min using an animal respirator (DW-3000A, Zhenhua, Henan, China). A left thoracotomy was done to expose the heart, and the left anterior descending (LAD) coronary artery was ligatured using a 6/0 Prolene suture (Jinhuan, Shanghai, China). A pale left ventricular myocardium indicates successful ligation. The ligature was removed after 0.5 h, followed by 24 h of reperfusion. A suture was passed through the LAD without being ligated in the sham group. BARD (Beyotime, Shanghai, China) was dissolved in DMSO. The stock solution was diluted in PBS to 0.25 mg/mL as the final concentration before intraperitoneal injection. Rats were intraperitoneally administered BARD or vehicle before I/R treatment, as shown in Figure 1(a).

### 2.2. Echocardiography

Cardiac function was measured using an echocardiography machine (Vevo2100, VisualSonics, Inc., Toronto, Canada). Rats were placed on a platform after being sedated with 2% isoflurane. Images were acquired using parasternal different views. Ejection fraction (EF) and fractional shortening (FS) were used as the primary indicators of cardiac function.

### 2.3. Measurements of Serum Markers

Blood samples were collected after 24 h reperfusion. The blood samples were placed at room temperature for 30 min and then centrifuged at 3000 rpm. The serum concentrations of creatine kinase (CK; cat. no. A032-1, Jiancheng, Nanjing), CK-MB (cat. no. E006-1, Jiancheng, Nanjing), troponin-I (cTnI; cat. no. H149, Jiancheng, Nanjing), and lactate dehydrogenase (LDH; cat. no. A020-2, Jiancheng, Nanjing) were measured in all groups using a biochemical analyzer.

### 2.4. Infarct Size

The 2,3,5-triphenyltetrazolium chloride (TTC) was used to measure the infarct size. Following anesthesia, the hearts were harvested, frozen for 30 min at -20°C, and then sectioned into 2 *μ*m slices (6 in total). Subsequently, the sections were incubated with 1% TTC solution, followed by fixation with 4% formalin. ImageJ was then used to analyze the area of infarction in each slice.

### 2.5. Myocardial Histopathological Staining

The harvested hearts were cut into slices, fixed in 4% formalin solution, dehydrated in alcohol and xylol, and then wrapped in paraffin max. Hearts were paraffin-embedded and cut into pieces for staining with H&E (cat. no. G1120, Solarbio) or Masson's trichrome (cat. no. KCD-R1022). An optical microscope (Olympus Corporation, Japan) was used to examine the stained sections.

### 2.6. TUNEL Staining

Myocardial apoptosis was observed with TUNEL staining using an apoptotic detection kit (cat. no. KCD-T1010; China). Following standard protocols, paraffin sections were dewaxed, hydrated, and incubated in proteinase K. After washing with PBS thrice, the samples were incubated in 3% hydrogen peroxide solution at room temperature for 20 min. After that, biotin-labeled solution was added to the samples, and then, the samples were incubated at 37°C for 60 min in the dark. Finally, the sections were stained with Streptavidin-HRP and DAB chromogenic fluid.

### 2.7. Detection of Malondialdehyde (MDA) and Superoxide Dismutase (SOD)

Myocardial tissues were lysed at -80°C, followed by centrifugation of the homogenate in accordance with the instructions. All the experimental steps were carried out exactly as specified in the product instructions. The activities of MDA and SOD were measured using assay kits (cat. no. A001-3 and A003-1, Jiancheng, Nanjing).

### 2.8. Immunohistochemistry

The paraffin sections of the heart were dewaxed and hydrated, followed by pretreatment with proteinase K for 20 min. Tissues were blocked using bovine serum albumin. The samples were incubated with anti-caspase-3 antibody (1 : 500; cat. no. Ab184787, Abcam), anti-8-OHdG antibody (1 : 100; cat. no. Ab48508, Abcam), anti-iNOS antibody (1 : 100; cat. no. Ab115819, Abcam), and anti-Nrf2 antibody (1 : 200; cat. no. Ab62352, Abcam) for overnight. Then, the secondary antibody was incubated for 60 min, followed by adding diaminobenzidine for incubation for 15 min. The tissue sections were examined using a microscope, and the images were processed using ImageJ software.

### 2.9. Western Blotting

Samples from myocardial tissues (20-40 mg) were placed on ice for 120 min with the RIPA cell lysate (cat. no. BL504, Biosharp) and protease inhibitors, followed by centrifugation. The supernatants were gathered. The protein concentrations were detected using a BCA protein assay kit (cat. no. BL521, Biosharp) and changed to 2 mg/mL for each specimen. Using sodium dodecyl sulfate-polyacrylamide gel electrophoresis (10% separation gel), the protein samples were divided; then, a polyvinylidene difluoride (PVDF) membrane was used for transfer. Five percentage of nonfat dry milk was used to block the membrane for 1 h. Subsequently, primary antibodies anti-Nrf2 (1 : 1000; cat. no. Ab62352, Abcam) and anti-HO-1 (1 : 2000; cat. no. Ab52947, Abcam) were added to the membrane for incubation overnight at 4°C. Being washed five times, the PVDF membrane was hatched with equivalent horseradish peroxidase-conjugated goat anti-rabbit secondary antibodies at room temperature for 1 h. Proteins were detected using ECL detection reagents (cat. no. BL520, Biosharp). Exposure was performed using a gel imaging system, and the results were calculated using ImageJ software.

### 2.10. Real-Time PCR

Following the manufacturer's protocol, a TRIzol reagent (cat. no. R401-01-AA, Vazyme) was used to extract total RNA from myocardial tissues. Total RNA (1 *μ*g) was synthesized to cDNA by reverse transcription using a cDNA synthesis kit (cat. no. MR101-02, Vazyme). Subsequently, cDNA was amplified using SYBR Green Master Mix. The target gene expression was normalized against *GAPDH*. The following primers were used: *Nrf2*, forward: CCCATTGAGGGCTGTGAT and reverse: TGTTGGCTGTGCTTTAGG; *HO-1*, forward: CGAAACAAGCAGAACCCA and reverse: CACCAGCAGCTCAGGATG.

### 2.11. Statistical Analysis

Analyses of data were executed through GraphPad Prism 9. Data values were presented as the mean ± standard deviation. In order to test the normal distribution, Shapiro-Wilk was applied. The equal variance was tested applying Levene's test, and unpaired *t*-test was employed to compare variables between two groups. Multiple analogies were compared by employing the one-way ANOVA and SNK test. *P* < 0.05 was judged as statistically significant.

## 3. Results

### 3.1. BARD Attenuates Myocardial I/R-Induced Cardiac Impairment

Cardiac function was assessed using echocardiography to explore the therapeutic outcomes of BARD on myocardial I/R injuries. The echocardiographic measurements, including EF and FS, are shown in Figure 1(b). The results indicated a significant decrease in EF (36.00 ± 8.24) and FS (17.71 ± 4.44) values of myocardial I/R rats. Interestingly, treatment with BARD significantly increased the values of EF (60.64 ± 3.79) and FS (32.89 ± 2.78) in rats suffering from myocardial I/R injury. Compared to the sham group, there were no obvious differences in the values of EF and FS of the sham plus BARD group (77.26 ± 4.06 and 46.80 ± 3.83 vs. 75.45 ± 3.54 and 44.95 ± 3.11, respectively).

Additionally, we demonstrated that the concentrations of CK, CK-MB, cTnI, and LDH were elevated following myocardial I/R but were decreased after BARD treatment (Figure 1(c)). Levels of CK (1.53 ± 0.40), CK-MB (2261.00 ± 95.66), cTnI (0.73 ± 0.10), and LDH (639.30 ± 52.36) were dramatically elevated in the I/R group compared to the I/R plus BARD group (0.86 ± 0.08, 1483.00 ± 63.07, 0.34 ± 0.05, and 398.90 ± 41.99, respectively). In the sham group, the levels of CK (0.35 ± 0.08), CK-MB (1021.00 ± 58.64), cTnI (0.15 ± 0.03), and LDH (179.60 ± 22.68) were similar to those in the sham plus BARD group (0.33 ± 0.07, 1038.00 ± 47.26, 0.14 ± 0.02, and 196.10 ± 23.03, respectively). These consequences revealed that myocardial I/R models were established successfully, and BARD can ameliorate myocardial I/R injuries.

### 3.2. BARD Reduces Myocardial Infarct Size and Decreases Cardiomyocyte Damage

**Figure 2:**
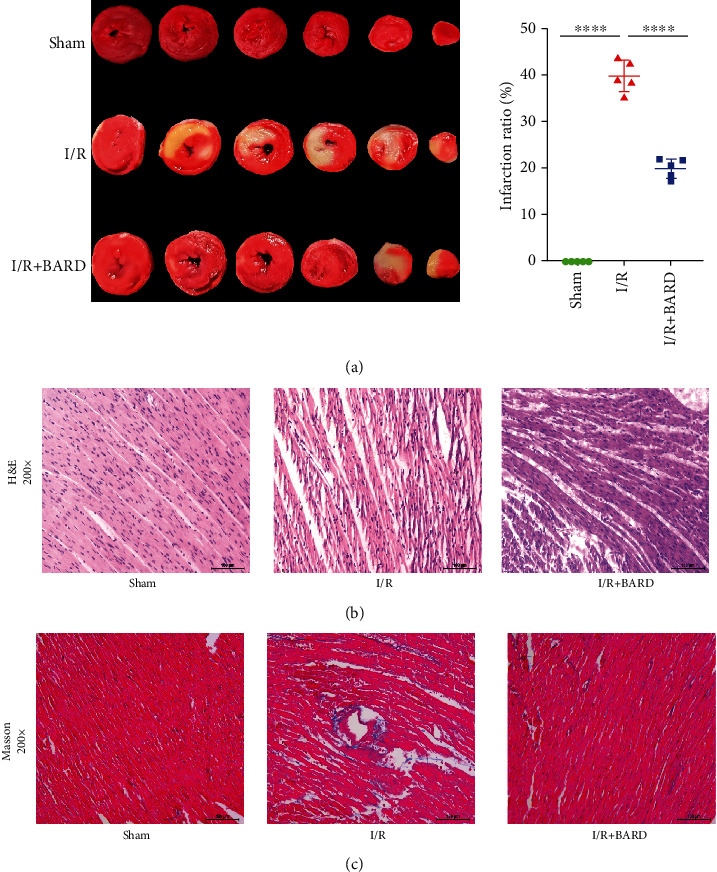
Bardoxolone methyl reduces the myocardial infarct size and decreases pathological changes in cardiomyocytes. (a) Triphenyltetrazolium chloride staining (TTC; *n* = 5), (b) H&E staining, and (c) Masson staining. Magnification of microscope: ×200. Scale bar: 100 *μ*m. ^∗∗∗∗^*P* < 0.0001.

Myocardial infarct size was measured by TTC staining after 24 h reperfusion. It was shown that myocardial I/R areas were 40.01 ± 3.35% in the I/R group and 20.04 ± 2.07% in the I/R plus BARD group, while no myocardial infarction was observed in the sham group (Figure 2(a)).

H&E staining was used to reveal the cardiomyocyte morphology. As manifested in Figure 2(b), in the sham group, cardiomyocyte structure was clear, intact, and well-arranged, and at the same time, there were no cardiomyocyte necrosis, collagen fiber proliferation, or inflammatory cell infiltration. In contrast, cardiomyocytes were disordered, arranged in mesophyll, and swollen in the I/R group, along with substantial inflammatory cell infiltration and collagen fiber hyperplasia. However, BARD treatment reduced cell loss and preserved the intact structure of most cardiomyocytes. Masson's staining examined myocardial collagen deposition in the myocardial interstitium. As illustrated in Figure 2(c), collagen fiber hyperplasia was remarkably elevated in the I/R group compared to the sham group. As expected, BARD treatment significantly attenuated the collagen fiber hyperplasia in the myocardial I/R rat model. These above results indicated that BARD relieved cardiac injuries.

### 3.3. BARD Prohibits Cardiomyocyte Apoptosis in Myocardial I/R

**Figure 3:**
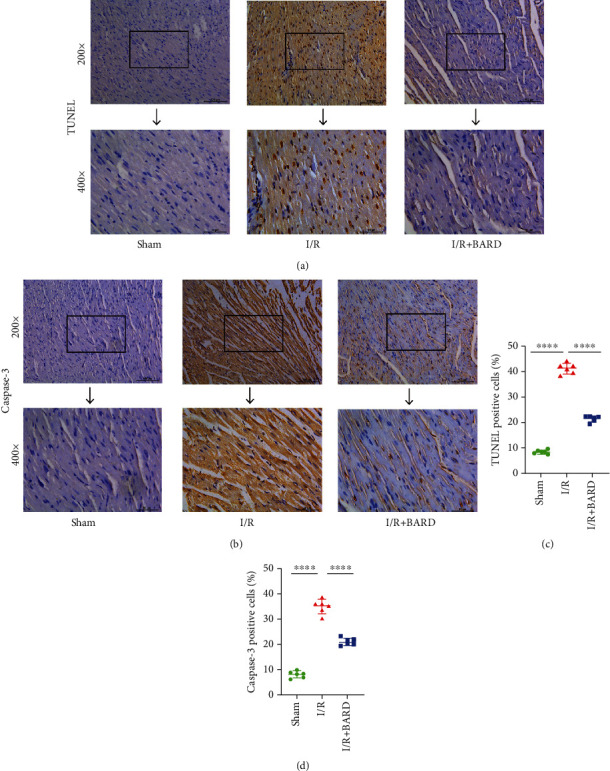
BARD alleviates cardiomyocyte apoptosis caused by myocardial I/R. (a) TUNEL staining. (b) Caspase-3 immunohistochemistry staining. (c) The positive cell amount of TUNEL (*n* = 6). (d) The amounts of caspase-3-positive cells (*n* = 6). Magnification of microscope: ×200 and ×400. Scale bar: 50 *μ*m and 100 *μ*m. ^∗∗∗∗^*P* < 0.0001.

TUNEL staining and immunohistochemical staining of caspase-3 were performed to evaluate the cardiomyocyte apoptosis. At high magnification, we observed clearly stained cardiomyocyte nuclei (Figure 3(a)). In the I/R group, apoptotic cardiomyocytes were round and atrophic, and nuclei were strongly stained. As illustrated in Figure 3(c), a minority of TUNEL-positive cells was found in the sham group as well as the I/R plus BARD group. Compared to the sham group (8.38 ± 0.77), the numbers of TUNEL-positive cells in the I/R group were raised obviously (41.32 ± 2.21). At the same time, treatment with BARD significantly decreased these cell numbers (21.49 ± 1.26).  Figure 3(b) shows caspase-3 immunohistochemical staining, and Figure 3(d) shows the amounts of caspase-3-positive cells. Compared to the sham group (8.11 ± 1.26), caspase-3 activity was elevated in the I/R group (35.14 ± 2.98). BARD treatment (21.19 ± 1.49) reduced the increase of caspase-3 activity caused by I/R. These results revealed that BARD could inhibit myocardial I/R-induced cardiomyocyte apoptosis.

### 3.4. BARD Alleviates Oxidative Stress in Myocardial I/R Injury

**Figure 4:**
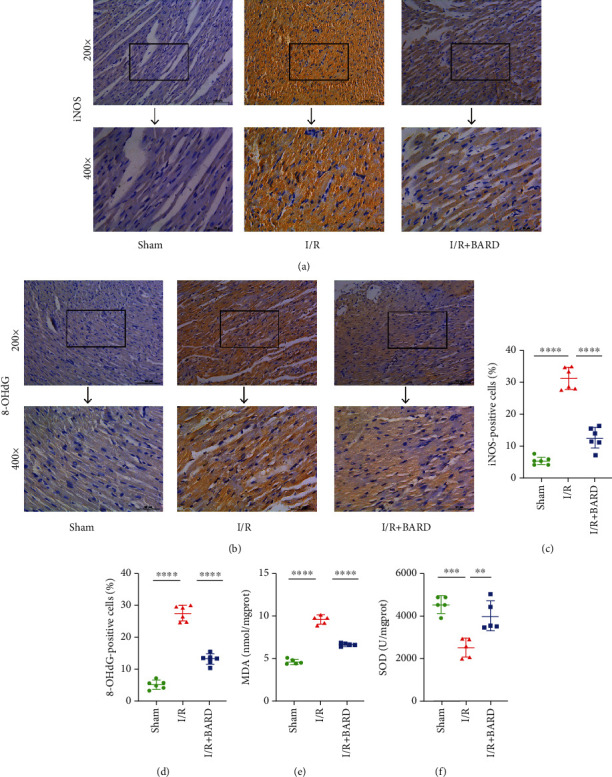
BARD alleviates oxidative stress-induced myocardial I/R. (a) iNOS immunohistochemistry staining. (b) 8-OHdG immunohistochemistry staining. (c) The amounts of iNOS-positive cells (*n* = 6). (d) The amounts of 8-OHdG-positive cells (*n* = 6). (e) MDA level (*n* = 5). (f) SOD content (*n* = 5). Magnification of microscope: ×200 and ×400. Scale bar: 50 *μ*m and 100 *μ*m. ^∗∗^*P* < 0.01, ^∗∗∗^*P* < 0.001, and ^∗∗∗∗^*P* < 0.0001.

The levels of iNOS, 8-OHdG, MDA, and SOD in heart tissues were detected to determine the effect of BARD on oxidative stress.  Figure 4(a) represents immunohistochemical staining of iNOS, while Figure 4(c) shows the numbers of iNOS-positive cells. We found that numbers of iNOS-positive cells (31.24 ± 3.52) in the I/R group were dramatically increased compared to those in the sham group (5.54 ± 1.27). Treatment with BARD decreased the amounts of iNOS-positive cells (12.65 ± 3.39). Figures 4(b) represents 8-OHdG immunohistochemical staining. As illustrated in Figure 4(d), the numbers of 8-OHdG-positive cells in the I/R group (27.20 ± 2.42) were remarkably increased in contrast to those in the sham group (5.31 ± 1.48). After BARD treatment, the numbers of positive cells (13.05 ± 1.61) were reduced. Additionally, we demonstrated that the I/R group had a higher MDA level (9.48 ± 0.53) than the sham group (4.56 ± 0.32) (Figure 4(e)). Treatment with BARD reduced the MDA levels induced by I/R (6.56 ± 0.20) (Figure 4(e)). As shown in Figure 4(f), the SOD activity (2488.00 ± 460.90) in the I/R group was significantly lower than that in the sham group (4523.00 ± 407.80), while treatment with BARD increased the SOD activity (3977.00 ± 708.50). These results implied that BARD ameliorated oxidative stress in the progression of myocardial I/R injury.

### 3.5. BARD Activates the Nrf2/HO-1 Pathway to Alleviate Myocardial I/R Injury

**Figure 5:**
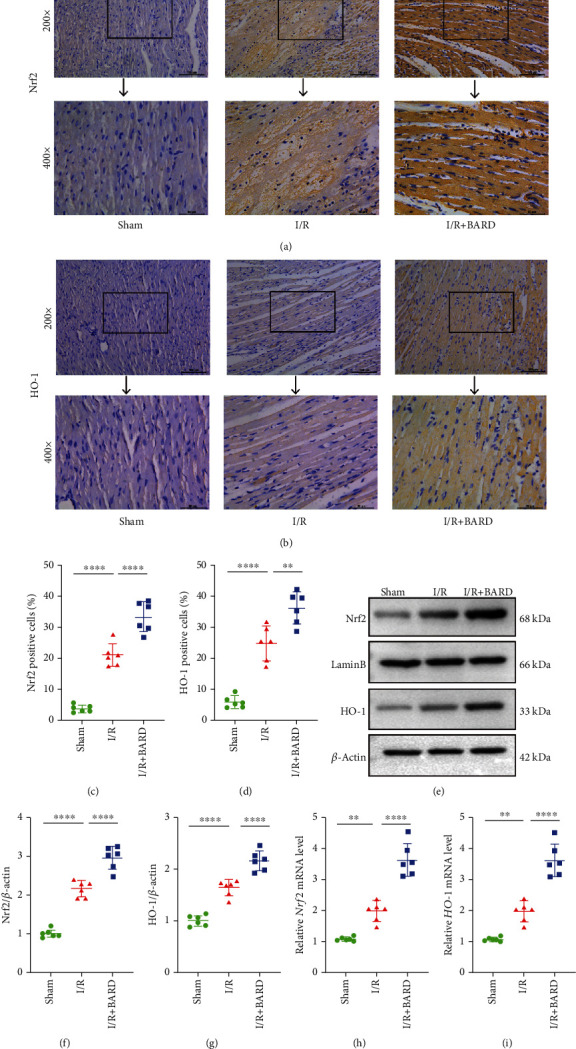
BARD activates the Nrf2/HO-1 pathway to alleviate myocardial I/R injury. (a) Nrf2 immunohistochemistry staining. (b) HO-1 immunohistochemical staining. (c) The positive cell amounts of Nrf2. (d) The amounts of HO-1-positive cells. (e) Western blot of Nrf2 and HO-1. (f, g) Nrf2 and HO-1 expression levels were determined by western blotting. (h, i) Nrf2 and HO-1 mRNA expression levels were measured by real-time PCR analysis. Magnification of microscope: ×200 and ×400. Scale bar: 50 *μ*m and 100 *μ*m. ^∗∗^*P* < 0.01 and ^∗∗∗∗^*P* < 0.0001.

The potential mechanisms of BARD in myocardial I/R injury were then ascertained.  Figure 5(a) shows immunohistochemical staining for Nrf2. The results indicated that the Nrf2 expression in the I/R group (20.86 ± 3.67) was distinctly increased relative to that in the sham group (3.68 ± 1.19) and was further elevated in the I/R plus BARD group (33.19 ± 4.92) (Figure 5(c)).

As shown in Figures 5(b) and 5(d), the amounts of HO-1-positive cells in the I/R group (25.00 ± 5.58) were markedly increased in contrast to those in the sham group (6.01 ± 1.94), while in the I/R plus BARD group (36.29 ± 5.16), the numbers of HO-1-positive cells were further boosted.

The protein levels of Nrf2 and HO-1 in myocardial samples were examined using western blotting (Figure 5(e)). As shown in Figures 5(f) and 5(g), the protein levels of Nrf2 (2.15 ± 0.20) and HO-1 (1.64 ± 0.15) in the I/R group were significantly enhanced compared with those in the sham group (1.00 ± 0.11 and 1.00 ± 0.10, respectively). As expected, both Nrf2 and HO-1 protein levels were further increased in the I/R plus BARD group (2.95 ± 0.29 and 2.16 ± 0.19, respectively).

Figures 5(h) and 5(i) show the mRNA expression of Nrf2 and HO-1. We indicated that the mRNA levels of Nrf2 in the I/R group (1.97 ± 0.33) were significantly elevated in contrast to those in the sham group (1.05 ± 0.07) and further increased in the I/R plus BARD group (3.60 ± 0.53). Similarly increasing trends were observed in the mRNA expression of HO-1, with the HO-1 mRNA expression of 1.05 ± 0.07, 1.97 ± 0.33, and 3.60 ± 0.53 in the sham, I/R, and I/R plus BARD groups, respectively. The above results revealed that BARD could activate the Nrf2/HO-1 pathway to be involved in regulating myocardial I/R injury.

## 4. Discussion

**Figure 6:**
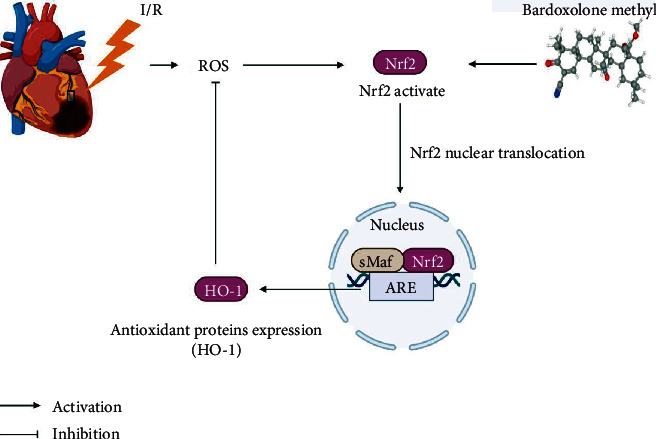
A graphical summary for the cardioprotective effect of bardoxolone methyl (BARD) against myocardial I/R injury.

In our study, we proved that BARD can protect rats from acute myocardial I/R injury effectively. Mechanistically, BARD promoted Nrf2 activation to initiate its nuclear translocation, facilitated the expression of HO-1, and inhibited cardiomyocyte apoptosis and oxidative stress, thereby reducing myocardial I/R injury (Figure 6).

AMI is typically caused by coronary artery stenosis. Coronary artery plaques can erode or rupture due to certain conditions. During this period, platelets are accumulated on the ruptured plaque to form a thrombus, thus blocking the lumen and resulting in myocardial ischemic necrosis [18]. In the 1960s, the main treatment for AMI was to prolong bed rest time and reduce physical activity. However, sudden death because of cardiac rupture, pericarditis, cardiogenic shock, and fatal arrhythmias was still common, and no effective measures were taken to reduce myocardial ischemia and necrosis [19]. Although the use of thrombolytic drug therapy, coronary intervention, and other strategies has significantly reduced the one-year mortality rate in recent decades, large-scale studies in real-world settings indicated that the overall mortality rate of AMI remains as high as 11% [20]. The recovery of normal perfusion after recanalization of the blocked coronary artery generally leads to myocardial I/R injury. The damage for myocardial tissues is further aggravated, which is closely related to the mortality of AMI. These public data indicated that alleviating myocardial I/R injury is able to effectively reduce myocardial infarction and AMI-associated mortality. Therefore, researchers have paid attention to the scheme of alleviating myocardial I/R injury.

The pathogenesis of I/R is complicated, including oxidative stress, calcium (Ca^2+^) overload, loss of mitochondrial membrane potential, endoplasmic reticulum stress, and inflammatory reactions [21–24]. The generation of mitochondrial ROS is considered as a key role in reperfusion injury [25]. During reperfusion, ROS accumulation can lead to DNA damage, lipid peroxidation, and protein dysfunction, thereby resulting in caspase-dependent apoptosis. Herein, treatment with BARD reduced the levels of myocardial MDA, iNOS, and 8-OHdG (biomarkers of lipid and DNA peroxidation) in the I/R rat model, suggesting that BARD could attenuate the lipid and DNA damage effectively. Furthermore, we observed that BARD increased SOD activity, indicating that BARD could reduce ROS accumulation and enhance cellular antioxidant capacity. Caspase-3, a key protein of the caspase family, can be activated by apoptotic signals to initiate the inactivation of some important downstream proteases and trigger cell apoptosis [26]. In this study, BARD treatment dramatically downregulated caspase-3 expression and improved TUNEL-staining results. The results implied that BARD can prohibit myocardial apoptosis.

Nrf2 is broadly expressed in the heart and considered as a key transcription factor associated with oxidative stress and inflammation [27, 28]. Nrf2 is generally maintained in the cytoplasm under nonstressful conditions but is transferred into the nucleus and triggers antioxidant genes under oxidative stress. HO-1, one of the majority target genes of Nrf2, can defend cells against oxidative stress and inflammation through heme catabolism [29]. Several previous reports have demonstrated that regulating the Nrf2/HO-1 pathway can reduce myocardial I/R injury. For instance, aloin can exert crucial antioxidant effects against myocardial I/R injury by regulating the Nrf2/HO-1 pathway [30]. Catalpol can suppress myocardial I/R damage by elevating the Nrf2/HO-1 expression [31].

BARD is a well-known drug that defends against oxidative stress. In 2021, the US Food and Drug Administration approved BARD as a new first-line oral treatment for patients with Alport syndrome. Although the BEACON study was discontinued earlier than expected because BARD was associated with serious heart failure, the subsequent analysis indicated that the increased risk of heart failure may be due to the fluid overload but not BARD application. Excluding the patients with high brain natriuretic peptide levels or a history of heart failure, the risk of cardiac failure was similar among BARD-treated patients and those who received the placebo (2%) [32]. Tian et al. have explored the association between low doses of BARD and heart failure and found that Nrf2 activation induced by BARD may improve heart failure following a myocardial infarction [33]. Through the activation of Nrf2, the expression of antioxidant enzymes is enhanced and the myocardial inflammation is decreased; thus, the oxidative stress is reduced and the cardiac function is improved. In this study, a rat model of myocardial I/R injury was constructed, and the therapeutic effect of BARD on myocardial I/R injury was ascertained for the first time. Similar to the previous studies, we also found that BARD treatment could improve cardiac output in myocardial I/R rats, and further studies discovered that BARD treatment markedly elevated the Nrf2 and HO-1 expression and improved the antioxidant capacity and histopathological properties in myocardial I/R rats, indicating the effectively therapeutic effect of BARD in myocardial I/R damage.

## 5. Conclusion

In summary, our study uncovers the protective role and regulatory mechanism of BARD in myocardial I/R injury, indicating that BARD can ameliorate oxidative stress and myocardial apoptosis in myocardial I/R injury rats via activating the Nrf2/HO-1 signaling pathway. These findings may contribute more information and insights on possible clinical therapeutic therapies for patients with myocardial I/R injury.

## Data Availability

The authors confirm that the data supporting the results of this study can be found in the article.
